# Evaluation of techniques for slice sensitivity profile measurement and analysis

**DOI:** 10.1120/jacmp.v15i2.4042

**Published:** 2014-03-06

**Authors:** Travis C. Greene, X. John Rong

**Affiliations:** ^1^ Department of Imaging Physics The University of Texas MD Anderson Cancer Center Houston TX USA

**Keywords:** slice sensitivity profile, quality assurance, computed tomography

## Abstract

The purpose of this study was to compare the resulting full width at half maximum of slice sensitivity profiles (SSP) generated by several commercially available point response phantoms, and determine an appropriate imaging technique and analysis method. Four CT phantoms containing point response objects designed to produce a delta impulse signal used in this study: a Fluke CT‐SSP phantom, a Gammex 464, a CatPhan 600, and a Kagaku Micro Disc phantom. Each phantom was imaged using 120 kVp, 325 mAs, head scan field of view, 32×0.625 mm helical scan with a 20 mm beam width and a pitch of 0.969. The acquired images were then reconstructed into all available slice thicknesses (0.625 mm−5.0 mm). A computer program was developed to analyze the images of each dataset for generating a SSP from which the full width at half maximum (FWHM) was determined. Two methods for generating SSPs were evaluated and compared by choosing the mean vs. maximum value in the ROI, along with two methods for evaluating the FWHM of the SSP, linear interpolation and Gaussian curve fitting. FWHMs were compared with the manufacturer's specifications using percent error and z‐test with a significance value of p<0.05. The FWHMs from each phantom were not significantly different (p≥0.089) with an average error of 3.5%. The FWHMs from SSPs generated from the mean value were statistically different $\left(p\leq 3.99\times 10^{13}\right)$. The FWHMs from the different FWHM methods were not statistically different (p≤0.499). Evaluation of the SSP is dependent on the ROI value used. The maximum value from the ROI should be used to generate the SSP whenever possible. SSP measurement is independent of the phantoms used in this study.

PACS number: 87.

## I. INTRODUCTION

Helical CT allows for the rapid and continuous volumetric scanning of patients by moving the patient through the gantry while tomographic data are acquired. The combination of tube rotation and table translation produces a dataset in which the projections wrap around the patient in a helix. In order for an axial slice to be reconstructed, the helical dataset needs to be interpolated to create a full set of projection data within the plane of reconstruction. The use of projection data from outside the reconstructed volume leads to a reduction in axial resolution^1^, ^2^, ^3^, ^4^, ^5^ The slice sensitivity profile (SSP) allows the measurement of the axial resolution of helical CT systems.

The SSP is generated by scanning a phantom containing a ball bearing, a thin film disk, or an angled wire.^5^, ^6^, ^7^, ^8^, ^9^ Regardless of the type of phantom, the phantom is designed to produce a point impulse resembling a delta function to the CT scanner in the cranial–caudal direction of the scanner. The imperfect response of the CT system blurs out the impulse function and produces a point spread function (PSF) in the axial plane.^6^, ^8^

There is a direct relationship between the thickness of a CT slice and the quality of the image that is produced. As the slice thickness increases, the contrast within the image may be reduced due to the increased probability of the image voxel containing different tissue types.^2^, ^3^ The resulting signal within the voxel will be the weighted average of the signal from the tissues within the voxel. The noise within the image will also be reduced as the image thickness increases due to an increase in the number of photons used to create the image. The increase in photons allows thicker slices to use a lower dose to achieve the same SNR. This relationship between slice thickness and image quality makes the SSP an important factor during CT protocol optimization. Furthermore, SSP measurement is a recommended part of CT acceptance testing, as indicated by the AAPM Report 39 and IEC 61223‐3‐5 reports.^10^, ^11^

During the infancy of Helical CT, much research was done to determine what factors of the CT scanner affected the axial resolution.^1^, ^2^, ^3^, ^4^, ^5^, ^6^, ^7^, ^8^, ^9^ The size of the focal spot, the width of the detectors, and the penumbra due to the focal spot are all factors that impact the SSP that are not controllable by the user.^1^, ^2^, ^3^, ^4^ There are robust data showing that adjustable factors affecting the sinogram data, such as pitch, detector configuration, and various methods of interpolating the sinogram data, namely full scan interpolation and half scan interpolation, impact the blurring of the system in the axial direction.^6^, ^8^, ^12^, ^13^ However, a robust procedure for SSP measurement in a clinical environment is not well defined. Concerns have been raised by clinical physicists regarding an appropriate procedure for SSP measurement. Specifically, are commonly available CT phantoms, such as the CatPhan or Gammex 464, acceptable for SSP measurement, or is a specialized SSP phantom required? Dose the image reconstruction interval impact the results? How should the reconstructed images be analyzed? The purpose of this study was to determine if any of these factors affect the measurement of the slice sensitivity profile, to develop an optimized procedure for acquiring phantom data, and to create an algorithm to efficiently analyze the images while reducing human error.

## II. MATERIALS AND METHODS

### A. Equipment

A GE CT scanner, Discovery CT750 HD (GE Healthcare, Waukesha, WI), was used to acquire all images used in this study. The detector assembly is comprised of 64 rows of 0.625 mm detector elements. The system is capable of scanning with two beam widths in helical mode, the widest radiation beam collimation is 40 mm (0.625 mm×64), and the smallest is 20 mm (0.625×32). Depending on the combinations of detector configuration and table speed, three selectable pitch factors for 20 mm beam configurations are 0.531, 0.969, and 1.375; and three selectable pitch factors for 40 mm beam configurations are 0.516, 0.984, and 1.375. The acquired data can be reconstructed into 0.625, 1.25, 2.50, 3.75, and 5.00 mm slices.^(14)^ Beam width and pitch are known to affect the width of the SSP; for simplicity, only the 20 mm beam collimation with a pitch of 0.969 was used in this study.^3^, ^4^, ^5^, ^6^, ^7^

Four phantoms were used in this study. The first phantom, CT‐SSP Phantom (Model 76‐412; Fluke Biomedical, Solon, OH), is specially designed to measure only the SSP of CT systems. It consists of a 0.25 mm acrylic bead that is embedded in a 2 cm diameter cylinder of low‐density foam. The test object is suspended in an acrylic cradle to enable easy positioning of the phantom test object at isocenter. The next two phantoms used in this study were designed for performance evaluation of a CT scanner system. They contain test objects for a variety of CT quality assurance tests including SSP analysis. The second phantom, ACR CT Accreditation Phantom (ACR CTAP) (Model 464; Gammex, Middleton, WI), is a 20 cm diameter Solid Water phantom that contains two 0.28 mm tungsten carbide beads that can be used to obtain the SSP at either isocenter or off isocenter locations. The third phantom used in the study, CatPhan Phantom (Model 600; The Phantom Laboratory, Salem, NY), is a 20 cm diameter phantom with the CTP591 module that contains two 0.28 mm tungsten carbide beads that can also be used to measure the SSP. The fourth phantom is a specialized SSP phantom that uses a thin film disc for the test object. The Micro Disc Phantom (Kyoto Kagaku, Torrance, CA) features a 1 mm diameter 0.05 mm thickness tungsten disc that is embedded in a 4 cm diameter cylinder of tissue‐equivalent plastic.

### B. Image acquisition

The signal produced by the phantom is blurred into several contiguous reconstructed images. Therefore, the PSF cannot be extracted from a single image. Accurate sampling of the PSF requires several overlapping slices that span a range large enough to fully encompass the range of blurring, usually 3‐5 times the thickness of the interrogated slice. Since the data in the sinogram contains projection data for the entire volume scanned, images can be reconstructed at arbitrary positions within the scanned volume.^6^, ^8^ To ensure that the SSP was adequately sampled, the images were reconstructed into overlapping slices using an increment equal to one‐tenth of the reconstructed slice thickness.

#### B.1 SSP dependency on phantoms

**Figure 1:**
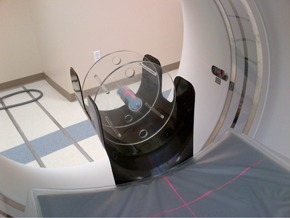
Phantom setup for the Fluke CT‐SSP Phantom with the test object positioned at isocenter.

The CT‐SSP Phantom was placed in the head holder and positioned at isocenter by adjusting the table position to align the target object using the alignment lasers to aid in positioning, as shown in Fig. 1. The CatPhan, ACR CTAP, and the Kagaku Micro Disc Phantoms were placed in the phantom holders supplied by their respective manufacturers. Each phantom was aligned to isocenter using the alignment lasers. Orthogonal scout scans were obtained to localize the SSP test object within the phantom. The table position was then adjusted to position the test object at isocenter. A second set of scout scans was then obtained to confirm that the test object was correctly positioned at isocenter.

To compare the results of SSP measurements from using different phantoms, the same phantom scan protocol was used in obtaining images, and the same method of image analysis was also applied for obtaining SSP profiles and, hence, the resulting full width at half maximum (FWHM) values. The phantom scan parameters are listed in Table 1. Each phantom was scanned using 120 kVp, 325 mAs, a 1.0 s rotation time, 20 mm z‐axis collimation, and a pitch of 0.969. The prescribed scan range was from I30 to S30 with the test object positioned at S0. This range allowed the SSP to be sampled ±5 reconstructed slice widths on either side of the slice positioned at isocenter. Each phantom scan was repeated five times to ensure reproducible results.

**Table 1:** Scan technique use to generate the SSP images

*Scan Type*	*Aperture*	*Pitch*	*SFOV*	*kVp*	*mA*	*Filter*	*Interpolation*
Helical 1.0 sec	20 mm	0.969:1	Head	120	325	Standard	Plus

The helical data obtained for each scan was then retrospectively reconstructed into 0.625 mm, 1.25 mm, 2.5 mm, and 5.0 mm slices using the Standard reconstruction algorithm with the Plus reconstruction option. To ensure adequate data sampling, the images were reconstructed using an increment equal to one‐tenth of the slice thickness. This sampling resulted in 101 images for each SSP that was measured. The prescribed settings are listed in Table 2.

**Table 2:** Reconstructed scan ranges and intervals used to generate SSP images

*Slice Thickness (mm)*	*Slice Interval (mm)*	*Start Position*	*End Position*	*Number of Images*
0.625	0.06	S3	I3	101
1.25	0.12	S6	I6	101
2.5	0.24	S12	I12	101
5.0	0.48	S24	I25	101

#### B.2 SSP dependence on scan FOV

The influence of the scan field of view (FOV) on the SSP measurement is not well documented in the literature. Changes in the scan FOV may alter the bowtie filter and change the noise properties of the images and, therefore, may impact the SSP. To evaluate the potential SSP dependence on scan FOV, the CT‐SSP Phantom was scanned using the head, small body, and large body field of views available on the CT scanner using our institution's standard procedure, described in 1, 2.

#### B.3 SSP dependence on data sampling interval

The increment between reconstructed slices has a direct impact on the sampling of the SSP. The accuracy of the SSP measurement depends on the shape of the profile curve and the ability to calculate the FWHM from the resulting curve. If undersampled, the accuracy of the FWHM measurement decreases. On the other hand, oversampling might only increase accuracy marginally. The increase in accuracy needs to be balanced with the space requirements for image storage and the time required for image transfer and analysis. To determine the appropriate data sampling interval, images were acquired using the CT‐SSP Phantom and then retro‐reconstructed with slice increments equivalent to the slice thickness divided by 10, 5, and 2.5, resulting in 101, 51, and 26 images, respectively.

### C. Image analysis

The SSP/PSF was created by reconstructing overlapping slices to adequately sample the function. A region of interest (ROI) was placed in the image containing the phantom object and propagated through all images. The signal value of the object in the ROI was plotted against the slice location producing the resulting SSP. The longitudinal resolution was then measured by taking the full width at half maximum (FWHM) of the SSP.^1^, ^2^, ^3^, ^4^, ^5^, ^6^, ^7^, ^8^, ^9^, ^10^, ^11^, ^12^, ^13^, ^14^

A program was developed for analysis of the reconstructed images to minimize inter‐SSP measurement variability. To accomplish this task, a commercially available, high level technical computing language (MATLAB R2009b; The MathWorks, Inc. Natick, MA) was used to create a program that analyzes the phantom images, generates a slice sensitivity profile, and determines the slice thickness by assessing the FWHM of the slice sensitivity profile.

The program allows the user to import a series of DICOM images, and apply the rescale slope and intercept from the DICOM header for accurate Hounsfield unit (HU) measurement.

**Figure 2:**
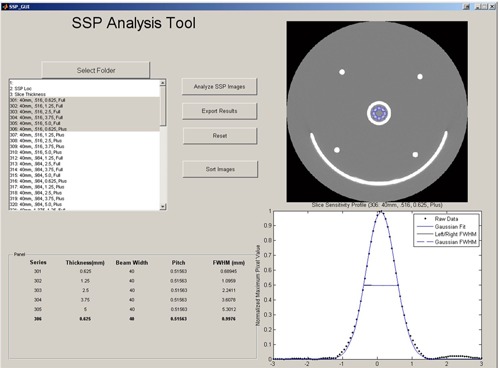
Graphical User Interface for the SSP analysis tool. This program allows the user to sort image files, import the phantom series, draw the ROI on the phantom, and measure the FWHM of the generated SSP.

An ROI was then drawn around the test object. The mean HU value and maximum HU value of the pixels within the ROI was then background‐corrected by obtaining an average HU value from the signal of the outermost images (images 1 and 101) and subtracting this value from each matrix element. This allowed for easier FWHM computation. The data were then normalized for uniform SSP plotting by dividing the matrix by the maximum element value. The program obtained the slice location from the DICOM header and stored the data as a 1×101 matrix. The SSP is generated by plotting the ROI data matrix against the slice location matrix. A Graphical User Interface was written to facilitate the use of the program, shown in Fig. 2.

#### C.1 SSP dependence on data sampling in a ROI

There are two approaches to generate a SSP from the measurement values of an ROI, namely the mean value from the ROI and the maximum value from the ROI. The program created two slice sensitivity profiles by plotting both the mean ROI value and the maximum ROI value against the slice location. The FWHM for each profile was obtained and compared with the manufacturer's specifications for each slice thickness to assess the impact of the ROI value on the accuracy of the measurement.

**Figure 3:**
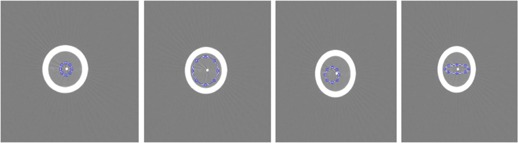
ROIs used to determine the impact of the ROI's size, shape, and position on the resulting SSP. Named from left to right: ROI1, ROI2, ROI3, and ROI4 for reference in results.

The program was developed such that the positioning of the ROI required user interaction. Therefore, it was necessary to determine if the characteristics of the ROI (size, position, shape) affected the resulting slice sensitivity profile. This was accomplished by assessing the FWHM of SSPs generated from four different ROIs, shown in Fig. 3.

#### C.2 SSP dependence on hw the half maximum was determined

During the initial creation of the program, it was found that the slice sensitivity profiles taken from the mean value in a ROI frequently did not have the same shape on the left versus right side of the SSP curve. Therefore, the following four methods for computing the FWHM were evaluated with both approaches of utilizing measurement values from a ROI — mean value and maximum value. They are: (1) determining the half‐maximum value based on the left side of the curve; (2) determining the half‐maximum value based on the right side of the curve; (3) determining the half‐maximum values for each side of the curve independently — combination of (1) and (2) above; (4) performing a Gaussian fitting on the data, since it has been shown that slice sensitivity profiles are Gaussian,^7^, ^8^ then determining the half‐maximum value based on the fitted Gaussian curve. The resulting slice thickness measurements of each method were compared with the manufacturer specifications to determine if the method for computing the FWHM of the SSP has an impact on the accuracy of the measured slice thickness.

## III. RESULTS

For all comparisons in this study, the manufacturer specified values for the desired slice thicknesses of 0.625 mm, 1.25 mm, 2.5 mm, and 5.0 mm are 0.98 mm, 1.38 mm, 2.88 mm, and 6.00 mm, respectively. Each comparison was repeated five times. The FWHM values listed in all tables represent the average of five FWHM measurements. Percent errors were computed from comparisons of measured FWHM values to those provided in the technical reference manual by the manufacturer.^(14)^

### A. data analysis dependencies

#### A.1 SSP dependence on hw the half maximum was determined

The average measured FWHM values of the five SSPs generated using the mean value from the ROI for each prescribed slice thickness are listed in Table 3. The measured FWHM values of the SSPs generated using the maximum value from the ROI are listed in Table 4. The results were obtained using the FWHM analysis methods 1‐4 described in the Materials and Methods section above.

**Figure 4:**
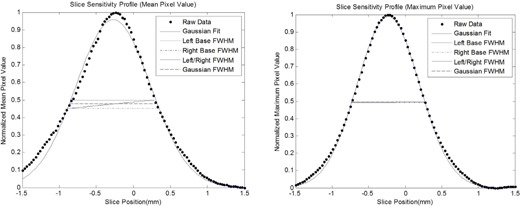
Graphs showing the difference between the FWHM measurements using the mean ROI value (left) vs. the maximum ROI value (right) to generate the slice sensitivity profile. The SSPs were generated using the Fluke SSP Phantom and ROI1.

When comparing the error of the FWHM value between the mean ROI generated SSP and maximum ROI generated SSP, the maximum ROI SSP produces FWHM values with a smaller percent error when compared to the manufacturer's specified values. For example, the mean value SSP measurement had a percent error of 20.0% when measuring the 0.625 mm slice using the Gaussian fit FWHM method, whereas the maximum value SSP measurement had an error of only 0.1%. The smaller standard deviation of the maximum value ROI measurements indicates that maximum ROI value SSPs have increased precision than mean ROI value SSPs. The z‐test resulted in a significant difference between the FWHMs from the mean ROI value SSPs $\left(p\leq 3.99\times 10^{13}\right)$. The FWHMs from the different FWHM methods were not statistically different when using the maximum ROI value SSPs (p≤0.499). Figure 4 illustrates the difference between the measured FWHM of the two SSPs, mean ROI value SSP and maximum ROI value SSP, for a 0.625 mm slice.

**Table 3:** The average measured slice thickness of five independently acquired SSPs obtained by using the mean value from the ROI. Slice thicknesses were obtained using four FWHM analysis methods

*Selected Thickness (mm)*	*0.625*	*1.25*	*2.50*	*5.00*
Manufacturer Spec. (mm)	0.98	1.38	2.88	6.00
	*Left Based FWHM*			
Measured FWHM (mm)	1.10	1.77	3.92	7.24
Error (mm)	0.12	0.39	1.04	1.24
Percent Error	12.5%	28.6%	36.1%	20.6%
Standard Deviation (mm)	0.094	0.174	0.237	0.385
	*Right Based FWHM*			
Measured FWHM (mm)	1.11	1.83	3.96	7.31
Error (mm)	0.13	0.45	1.08	1.31
Percent Error	13.4%	32.5%	37.4%	21.9%
Standard Deviation (mm)	0.086	0.253	0.355	0.397
	*Two Sided FWHM*			
Measured FWHM (mm)	1.12	1.81	3.93	7.19
Error (mm)	0.14	0.43	1.05	1.19
Percent Error	13.8%	30.9%	36.4%	19.8%
Standard Deviation (mm)	0.076	0.210	0.302	0.384
	*Gaussian Fit FWHM*			
Measured FWHM (mm)	1.18	1.82	3.95	7.29
Error (mm)	0.20	0.44	1.07	1.29
Percent Error	20.0%	31.8%	37.3%	21.6%
Standard Deviation (mm)	0.086	0.153	0.296	0.374

**Table 4:** The average measured slice thickness of five independently acquired SSPs obtained by using the maximum value from the ROI. Slice thicknesses were obtained using four FWHM analysis methods

*Selected Thickness (mm)*	*0.625*	*1.25*	*2.50*	*5.00*
Manufacturer Spec. (mm)	0.98	1.38	2.88	6.00
	*Left Based FWHM*			
Measured FWHM (mm)	0.97	1.38	2.98	6.23
Error (mm)	−0.01	0.00	0.10	0.23
Percent Error	1.1%	0.3%	3.6%	3.8%
Standard Deviation (mm)	0.015	0.021	0.041	0.079
	*Right Based FWHM*			
Measured FWHM (mm)	0.97	1.40	3.05	6.35
Error (mm)	−0.01	0.02	0.17	0.35
Percent Error	0.7%	1.4%	5.8%	5.8%
Standard Deviation (mm)	0.016	0.018	0.055	0.146
	*Two Sided FWHM*			
Measured FWHM (mm)	0.97	1.39	3.02	6.29
Error (mm)	−0.01	0.01	0.14	0.29
Percent Error	0.9%	0.6%	4.7%	4.8%
Standard Deviation (mm)	0.016	0.019	0.046	0.113
	*Gaussian Fit FWHM*			
Measured FWHM (mm)	0.98	1.39	3.04	6.43
Error (mm)	0.00	0.01	0.16	0.43
Percent Error	0.1%	0.7%	5.6%	7.1%
Standard Deviation (mm)	0.016	0.020	0.035	0.083

#### A.2 SSP dependence on data sampling in a ROI

The resulting FWHM's of the slice sensitivity profiles generated from the four ROIs shown in Fig. 3 are listed in Table 5. For simplicity, these values were obtained using the Gaussian fit method #4 only. The percent errors ranged between 2.0% when measuring 0.625 mm slices to 6.3% when measuring 5 mm slices. These measurements were not statistically different from the manufacturer's results (p≥0.886).

**Table 5:** Measured slice thicknesses for SSPs generated from the maximum ROI value. The FWHM values were obtained by fitting a Gaussian curve to the SSP ata

*Selected Thickness (mm)*	*0.625*	*1.25*	*2.50*	*5.00*
Manufacturer Spec. (mm)	0.98	1.38	2.88	6.00
	*ROI1*			
Max ROI Value (mm)	1.00	1.42	3.02	6.38
Standard Deviation (mm)	0.009	0.009	0.007	0.006
Error (mm)	0.02	0.04	0.14	0.38
Percent Error	2.1%	2.6%	5.0%	6.3%
	*ROI2*			
Max ROI Value (mm)	1.00	1.39	2.98	6.26
Standard Deviation (mm)	0.008	0.053	0.027	0.016
Error (mm)	0.02	0.01	0.10	0.26
Percent Error	2.0%	1.0%	3.5%	4.3%
	*ROI3*			
Max ROI Value (mm)	1.00	1.41	3.02	6.35
Standard Deviation (mm)	0.066	0.081	0.072	0.073
Error (mm)	0.02	0.03	0.14	0.35
Percent Error	2.5%	2.4%	4.8%	5.8%
	*ROI4*			
Max ROI Value (mm)	1.00	1.41	3.00	6.29
Standard Deviation (mm)	0.018	0.083	0.115	0.048
Error (mm)	0.02	0.03	0.12	0.29
Percent Error	2.0%	2.4%	4.2%	4.8%

### B. Data acquisition dependencies

#### B.1 SSP dependency on phantoms

The measured FWHM for each of the four phantoms scanned is shown in Table 6. The data analysis for the data acquisition dependencies was performed only on the SSPs generated from the maximum value from the ROI using the Gaussian fit method to calculate the FWHM. The maximum difference in the percent error between the four phantoms was 2.2%, 2.1%, 6.3%, and 8.7% for the 0.625 mm, 1.25 mm, 2.5 mm, and 5.0 mm slices, respectively. The CatPhan Model 500 produced the most accurate measurements, while the Fluke CT‐SSP phantom produced the most precise measurements. These measurements were not statistically different from the manufacturer's results for all phantoms (p≥0.089).

**Table 6:** Measured FWHM of slice sensitivity profiles using four point response phantoms. The SSPs were generated using the maximum ROI value plotted against the slice location. The FWHM analysis was performed by fitting a Gaussian curve to the data and taking the FWHM of the Gaussian curve

*Selected Thickness (mm)*	*0.625*	*1.25*	*2.50*	*5.00*
Manufacturer Spec. (mm)	0.98	1.38	2.88	6.00
	*Fluke CT‐SSP Phantom*			
Measured FWHM (mm)	1.00	1.41	3.02	6.39
Error (mm)	0.02	0.03	0.14	0.39
Percent Error	2.2%	2.1%	4.9%	6.5%
Standard Deviation (mm)	0.002	0.005	0.009	0.018
	*Gammex 464 (ACR Phantom)*			
Measured FWHM (mm)	0.98	1.39	3.04	6.43
Error (mm)	0.00	0.01	0.16	0.43
Percent Error	0.1%	0.7%	5.6%	7.1%
Standard Deviation (mm)	0.016	0.020	0.035	0.083
	*CatPhan Model 500*			
Measured FWHM (mm)	0.97	1.38	2.98	6.31
Error (mm)	−0.01	0.00	0.10	0.31
Percent Error	0.7%	0.03%	3.5%	5.2%
Standard Deviation (mm)	0.009	0.017	0.014	0.115
	*Kagaku Phantom*			
Measured FWHM (mm)	0.99	1.39	3.06	6.52
Error (mm)	0.01	0.01	0.18	0.52
Percent Error	1.0%	0.9%	6.3%	8.7%
Standard Deviation (mm)	0.016	0.014	0.017	0.048

#### B.2 SSP dependency on scan FOV

The FWHM of the SSPs using different scan field of views using the CT‐SSP Phantom are shown in Table 7. The maximum difference in percent error was 2.9%, 2.1%, 5.3%, and 6.5% for the 0.625 mm, 1.25 mm, 2.5 mm, and 5.0 mm slices, with the large body scan FOV producing the most accurate results overall. However, the head scan FOV produced the most precise results. There was no statistical difference (p≥0.097) between the FWHM datasets generated from the three scan FOVs.

**Table 7:** Measured FWHM of slice sensitivity profiles using the CT‐SSP Phantom. The SSPs were generated using the maximum ROI value plotted against the slice location. The FWHM analysis was performed by fitting a Gaussian curve to the data and taking the FWHM of the Gaussian curve

*Selected Thickness (mm)*	*0.625*	*1.25*	*2.50*	*5.00*
Manufacturer Spec. (mm)	0.98	1.38	2.88	6.00
	*Head*			
Measured FWHM (mm)	1.00	1.41	3.02	6.39
Error (mm)	0.02	0.03	0.14	0.39
Percent Error	2.2%	2.1%	4.9%	6.5%
Standard Deviation (mm)	0.002	0.004	0.009	0.018
	*Small Body*			
Measured FWHM (mm)	1.00	1.39	3.03	6.27
Error (mm)	0.02	0.01	0.15	0.27
Percent Error	1.8%	1.1%	5.3%	4.6%
Standard Deviation (mm)	0.003	0.005	0.010	0.043
	*Large Body*			
Measured FWHM (mm)	1.01	1.41	2.98	6.01
Error (mm)	0.03	0.03	0.10	0.01
Percent Error	2.9%	1.8%	3.5%	0.2%
Standard Deviation (mm)	0.004	0.005	0.038	0.172

#### B.3 SSP dependence on data sampling interval

The FWHM for the SSPs generated using different sampling intervals are shown in Table 8. The maximum difference in percent error was 2.2%, 2.6%, 5.0%, and 6.5% for the 0.625 mm, 1.25 mm, 2.5 mm, and 5.0 mm slices. Overall, a sampling interval equal to one‐tenth of the slice thickness produced the most accurate and most precise results. However, there was no statistical difference between the measured FWHM values and the manufacturer's specifications for each data sampling interval (p≥0.253).

**Table 8:** Measured FWHM of slice sensitivity profiles using the CT‐SSP Phantom. The SSPs were generated using the maximum ROI value plotted against the slice location. The FWHM analysis was performed by fitting a Gaussian curve to the data

*Selected Thickness (mm)*	*0.625*	*1.25*	*2.50*	*5.00*
Manufacturer Spec. (mm)	0.98	1.38	2.88	6.00
	Interval=Slice Thickness/10			
Measured FWHM (mm)	1.00	1.41	3.02	6.39
Error (mm)	0.02	0.03	0.14	0.39
Percent Error	2.2%	2.1%	4.9%	6.5%
Standard Deviation (mm)	0.002	0.005	0.009	0.018
	Interval=Slice Thickness/5			
Measured FWHM (mm)	1.00	1.41	3.02	6.41
Error (mm)	0.02	0.03	0.14	0.41
Percent Error	2.3%	2.6%	5.0%	6.9%
Standard Deviation (mm)	0.002	0.005	0.012	0.042
	Interval=Slice Thickness/2.5			
Measured FWHM (mm)	1.00	1.41	2.99	6.42
Error (mm)	0.02	0.03	0.11	0.42
Percent Error	2.6%	2.3%	4.9%	7.1%
Standard Deviation (mm)	0.002	0.005	0.012	0.041

## IV. DISCUSSION

There are fundamentally two methods for determining the SSP for a given set of point response phantom images. One method is to use the mean signal value from the ROI and the other is to use the maximum value from the ROI. Using the manufacturer‐specified values as the gold standard, our measurements show that SSPs generated from the maximum ROI value are more accurate than the SSPs generated from the mean ROI value.^(9)^ Since the methodology the manufacturers used to generate the specifications is unknown, the better agreement between our measurements and theirs may be due to a coincidence between our testing methodology and that of the manufacturers. However, our data show the FWHMs taken from the mean value SSPs were larger than those taken from the maximum value SSPs in all cases measured. Furthermore, the accuracy is improved as the area of the ROI decreases. The reduction in the number of pixels within the ROI causes the mean value of the ROI to approach the maximum value of the ROI.

Our results are primarily based on fitting a Gaussian curve to the data and measuring the slice thickness by taking the FWHM of the Gaussian curve. Each test performed in this study also had the FWHM calculated by linear interpolation of the data. The half‐maximum value was chosen by using the left side minimum, right side minimum, and using both left and right side to estimate the slice position of the half‐maximum value. Overall, the accuracy of the four methods used to calculate the FWHM of the SSP were similar, but only when using the maximum value from the ROI. The FWHM values from the Gaussian fit method showed increased precision when measuring SSPs generated from the same scan parameters. The Gaussian fit method also showed less variation when measuring SSPs generated from reduced sampling intervals. Therefore, the Gaussian fit method was chosen to compute all the FWHM measured in this study due to this increased precision.

Prior research has shown that a point response phantom is the preferred choice when measuring a multislice helical SSP.^(7)^ Three of the phantoms used in this study have a high attenuating bead with a diameter smaller than the half of the width of the detector elements of the scanner (0.625 mm). The fourth phantom contained a thin film disk (0.05 mm) as the test object. Our results did not show a correlation between the accuracy of the FWHM measurement and the phantom used to obtain the SSP. This suggests that any of the point response phantoms evaluated in this study will produce an accurate point spread function and do not pose the superiority of these phantoms over other available phantoms containing similar test objects.

At our facility we have use the Fluke CT‐SSP Phantom to measure the SSP uring the acceptance testing of six GE Discovery CT750 HD systems. The phantom was scanned using the head SFOV, 120 kVp, and 250 mAs. The images were analyzed using SSPs generated from the maximum ROI value. The FWHM was evaluated using the Gaussian fit method. The acceptance testing includes all beam width, pitch, and reconstruction type (Full, Plus) combinations for each available image thicknesses (0.625 mm, 1.25 mm, 2.5 mm, 3.75 mm, and 5.0 mm). We have found good agreement between the measured FWHM values and the manufacturer's specifications. Typical errors have been in the 2%‐4% range, with the overall difference in the value being less than 0.5 mm in most cases. Larger errors were common with the 5.0 mm slices.

## V. CONCLUSIONS

The results indicate that any of the four commercially available phantoms evaluated in this study are appropriate for the measurement of the SSP of a CT system. The results indicate that all of the tested image reconstruction intervals produced FWHM values with equivalent accuracy, but the precision decreased as the number of data samples decreased. Finally, our results indicate that either linear interpolation or Gaussian fit methods for analyzing the FWHM will produce reliable results when assessing the slice width. However, it is critical that the SSP be generated from the maximum value in the ROI when possible, or a small ROI is used when only the mean value can be obtained.
